# Fabrication of Perfusable Vascular Channels and Capillaries in 3D Liver-like Tissue

**DOI:** 10.1038/s41598-020-62286-3

**Published:** 2020-04-14

**Authors:** Nobuhito Mori, Yuka Akagi, Yukiko Imai, Yuzo Takayama, Yasuyuki S. Kida

**Affiliations:** 1 Biotechnology Research Institute for Drug Discovery, National Institute of Advanced Industrial Science and Technology (AIST), Central 5-41, 1-1-1 Higashi, Tsukuba, Ibaraki 305-8565 Japan; 2 Advanced Photonics and Biosensing Open Innovation Laboratory, National Institute of Advanced Industrial Science and Technology (AIST), Central 5-41, 1-1-1 Higashi, Tsukuba, Ibaraki 305-8565 Japan; 3 Department of Plastic and Reconstructive Surgery, University of Tsukuba, 1-1-1 Tennodai, Tsukuba, Ibaraki 305-8577 Japan

**Keywords:** Assay systems, Regenerative medicine, Tissue engineering

## Abstract

Although various production methods for 3D vascularised tissues have been developed, constructing capillary-like structures branching from perfusable large channels remains difficult. This study describes a method to fabricate tube-shaped 3D liver-like tissue (tubular liver tissue) with large channels and capillary-like structures using a perfusion device. The perfusion device functions as an interface between the tissue and an external pump, as it has connectors equipped with anchors that hold the tissue in response to its shrinkage, which is accompanied by the self-organisation of capillary-like structures. Histological analysis revealed that perfusion via the large channel induced capillary formation around the channel and maintained proper tissue functions. Accompanied by structural examinations, global gene expression analysis supported this finding; specifically, genes involved in angiogenesis were enriched in the perfused condition. Furthermore, we confirmed the penetrability of the capillary-like structures by infusing India ink, as well as substance exchange by measuring the amounts of secreted albumin. These lines of evidence indicate that our method can be used to construct 3D tissues, which is useful for fields of *in vitro* tissue regeneration for drug development and regenerative medicine.

## Introduction

Organ functions are maintained by vascular systems. For example, the liver receives blood from two large vessels, the hepatic artery and portal vein. Blood from the hepatic artery is sent from the heart and contains abundant oxygen, whereas that from the portal vein contains nutrition and xenobiotics including drugs that are mainly absorbed in the small intestine. The blood from these vessels merges and flows through capillaries of the liver called sinusoids. Through the sinusoids, oxygen, nutrients, and xenobiotics are provided to hepatocytes lined around the sinusoids, and then these are consumed and metabolised. Consequently, these metabolites flow into the central vein. Thus, the vascular system has important roles in organ functions.

The vascular system is also important for artificial 3D tissues, which have attracted considerable attention as a tool for drug testing and as grafts for transplantation in regenerative medicine. Since the diffusion of substances including nutrients and oxygen is limited in monolithic 3D tissue, capillary-like structures have been developed to maintain viability and functionality utilising self-organising endothelial cells^1–3^. Recently, a promising method to construct vascularised tissue with a high density of parenchymal cells was reported^4^; here, parenchymal cells and endothelial cells are co-cultured with mesenchymal cells that induce tissue compaction and the self-organisation of capillary-like structures. Although this method exploits the spontaneous formation of capillary-like structures by cells, it is essential to construct additional large channels with branching capillary-like structures to perform perfusion and promote substance exchange (i.e. nutrient and oxygen uptake, secretion of cellular products) *in vitro*. However, it is thought that the construction of large channels and the maintenance of structure and function are difficult using conventional methods^5–8^.

In this study, we aimed to overcome difficulties such as the collapse or detachment of large channels from the perfusion system, which are caused by tissue shrinkage, by designing anchor structures and using our fabricated perfusion device. Using this device, we fabricated a tube-shaped 3D liver tissue (tubular liver tissue) containing capillary-like structures branched from the large channel. The tubular liver tissue was maintained by the device despite shrinkage. Here, we also examined the functional advantages of the perfusable tubular liver tissue.

## Results

### Fabrication of tubular liver tissue

**Figure 1:**
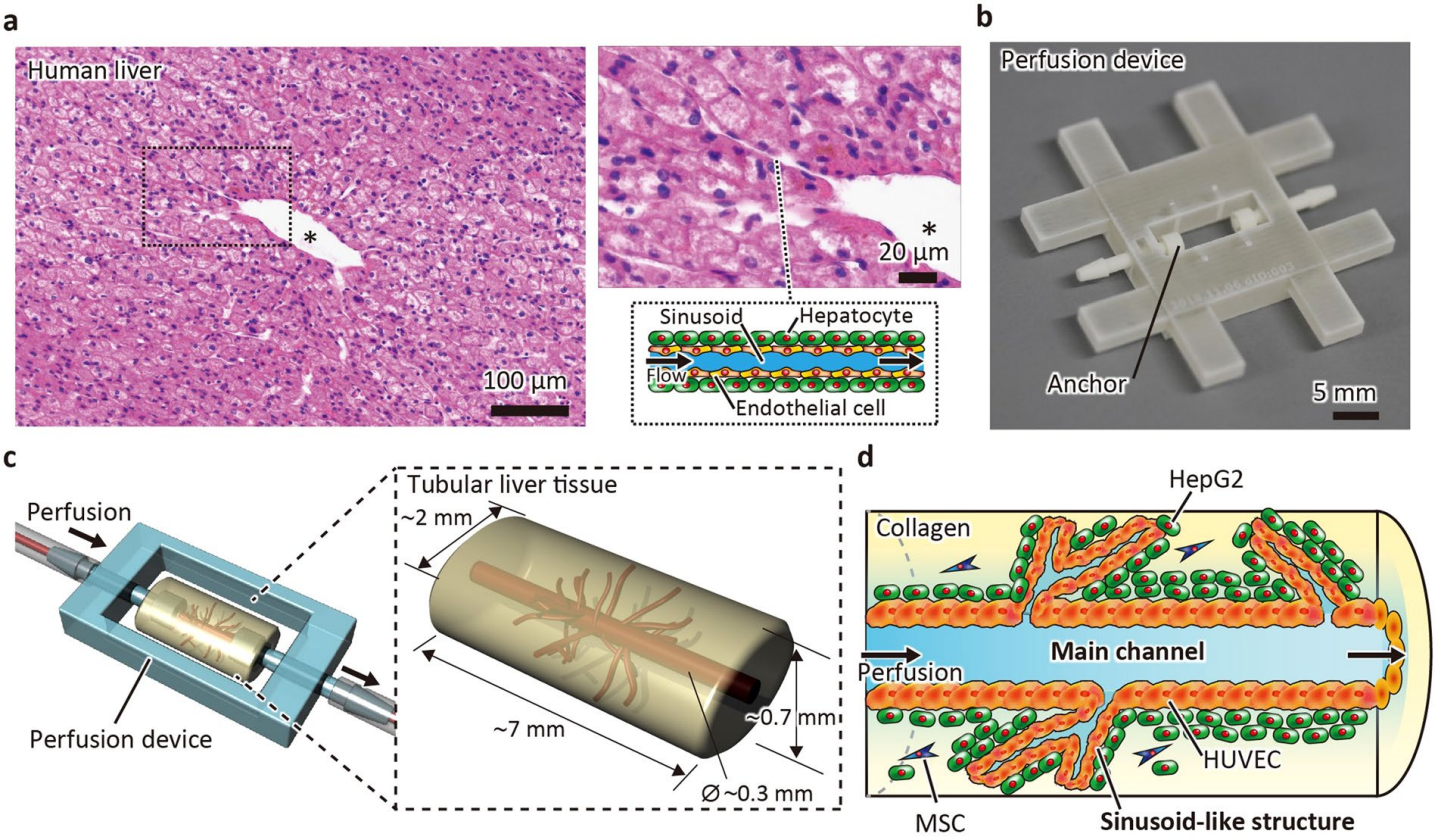
Schematic of a tubular liver tissue enhanced with a large main channel and capillary-like structures. (**a**) HE-stained section of the human liver. The section shows a large vascular channel (indicated by asterisk) and sinusoids lined by endothelial cells and surrounded by hepatocytes. (**b**) The perfusion device. The device has connectors equipped with an anchor that fixes the tissue. (**c**) The liver tissue fabricated in a perfusion device. (**d**) Longitudinal section of the liver tissue. The liver tissue is composed of a collagen gel populated with HepG2 cells, human umbilical vein endothelial cells (HUVECs), and mesenchymal stem cells (MSCs), and has a main channel connected with sinusoid-like structures.

The human liver has a vascular system that contains large vessels such as the hepatic artery, portal vein, and central vein, as well as sinusoids (Fig. 1a). To mimic the precise structure of the liver and sinusoids, a self-organisation process including compaction and vascularisation is one of most promising methods^4^. According to a previous report, the minimum components required to construct tissue with capillary-like structures include parenchymal cells, endothelial cells, and mesenchymal stem cells (MSCs) populated in the ECM substrate^4^. Thus, we attempted to fabricate a liver-like tissue composed of a collagen gel populated with hepatocellular carcinoma cells (HepG2 cells), human umbilical vein endothelial cells (HUVECs), and MSCs. To fabricate and maintain a large channel (main channel), we used a perfusion device that has connectors equipped with anchors to hold the tissue in response to shrinkage; this was originally invented for skin tissue, which also shrinks during cultivation^9,10^. The device was also treated with air plasma and fibronectin to increase the adhesion of collagen and cells. According to this strategy, we designed tubular liver tissue with a morphology similar to that of the human liver (Fig. 1b–d). The sinusoid-like structures self-organised from the cells were connected to the main channel that was perfusable due to the implementation of a perfusion device.

**Figure 2:**
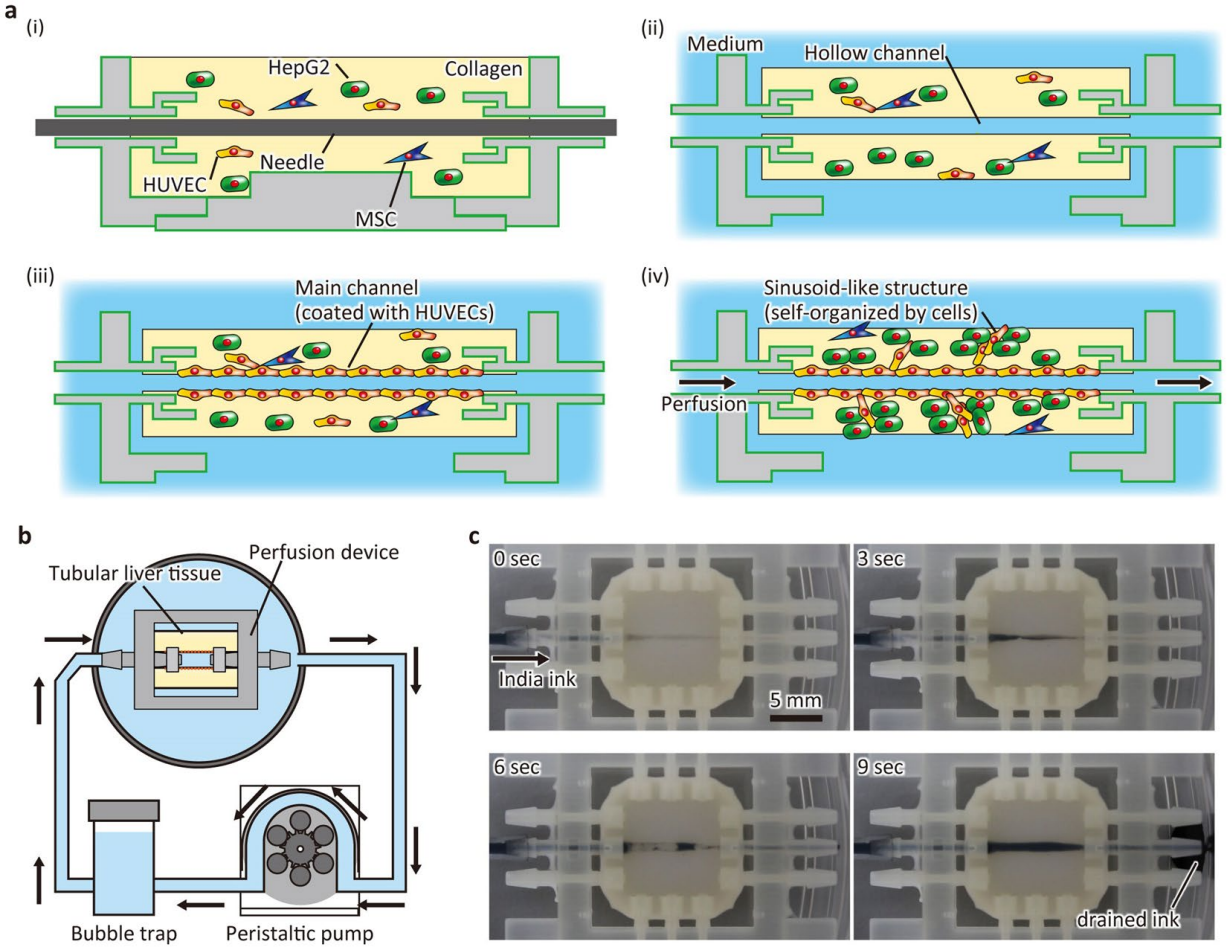
Fabrication process of liver tissue. (**a**) Diagram showing the construction process. (i) The cell-populated collagen gel is filled in the device. (ii) A needle is extracted to make a hollow channel. (iii) The hollow channel is coated with human umbilical vein endothelial cells (HUVECs). (iv) The tissue is perfused with medium. MSC, mesenchymal stem cell. (**b**) Perfusion system. The tissue is perfused using a peristaltic pump. A bubble trap is set to remove bubbles generated during circulation. (**c**) Sequential images of perfusion test. India ink was infused into the tissue and drained from the outlet.

The liver tissue was fabricated as shown in Fig. 2a. Briefly, collagen solution with a suspension of HepG2 cells, HUVECs, and MSCs was poured into the device, in which the surface was treated with air plasma and fibronectin (Fig. 2a-i). The initial cell density was in the order of 10^7^ cells/mL, which was designed to obtain the physiologically-relevant density (~10^8^ cells/mL) after shrinkage. The ratio of cells was determined based on previous reports^3,11^. After collagen gelation, a hollow channel was constructed via needle extraction (Fig. 2a-ii). Subsequently, HUVECs were infused and inoculated onto the wall of the hollow channel (Fig. 2a-iii). Then, the constructed main channel was perfused with medium at 1 mL/hour using a peristaltic pump (Fig. 2a-iv,b). The flow rate was determined such that the shear stress was in the order of 10^−1^ dyn/cm^2^, which is comparable to the shear stress of the liver^12^. During the culture period, the tissue did not detach from the device, although it shrunk due to cellular traction forces. We performed perfusion for up to 8 days using the system, while the main channels were occasionally clogged by chance. To show that the main channel could be perfused, India ink was infused after 4 days of perfusion culture (Fig. 2c, Movie S1). The infused ink flowed into the main channel of the tissue from the inlet connector of the device and flowed through the main channel. The infused ink was then drained from the outlet connector, and no leakage was observed at the interface between the tissue and connector. These results indicate that the device can hold the liver tissue against the pressure of the medium flow and tensile stress induced by tissue shrinkage due to the anchor and surface treatment.

### Histological analysis shows the formation of sinusoid-like structures induced by perfusion

**Figure 3:**
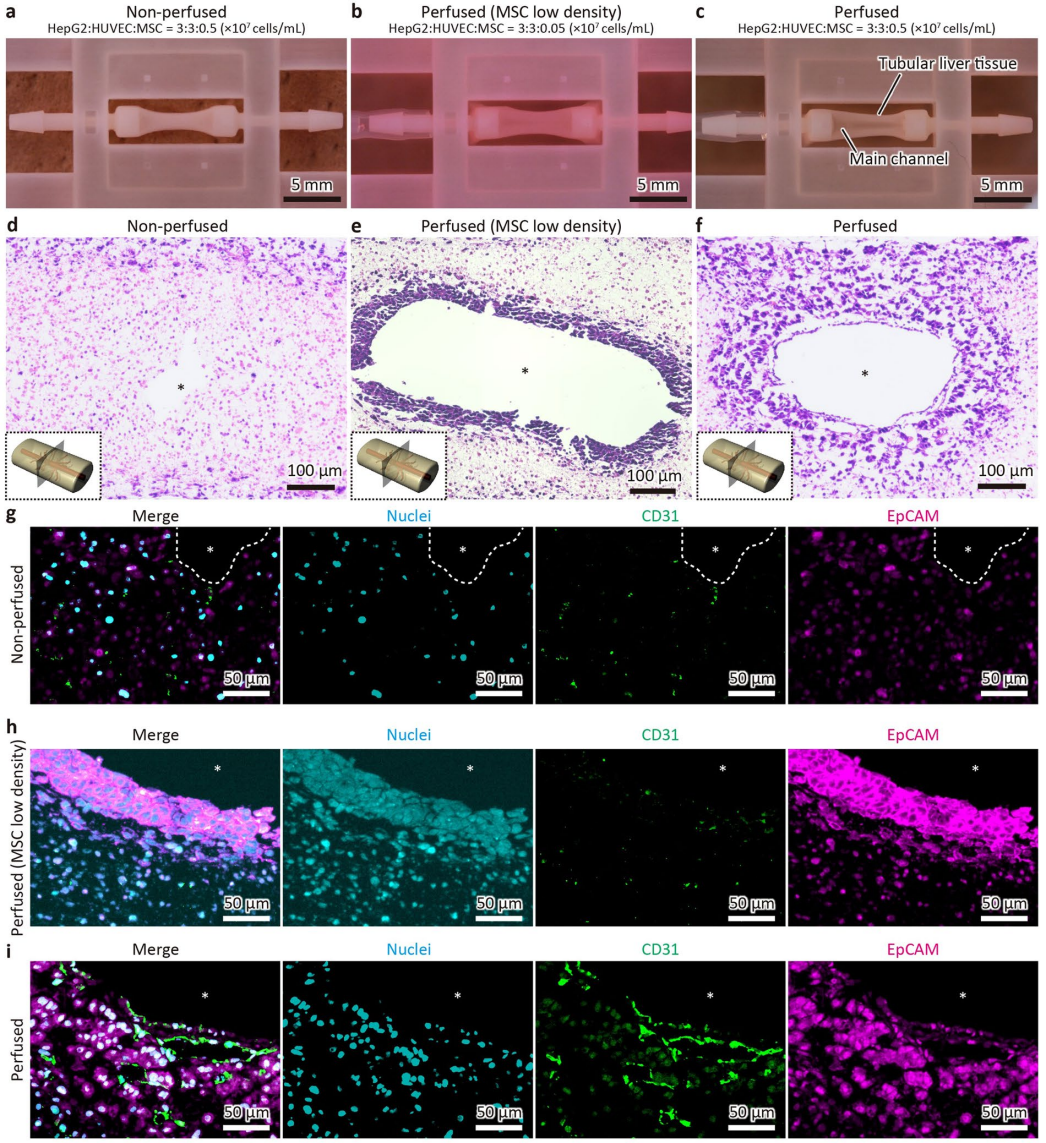
Histological analysis of perfused liver tissues. (**a–c**) Appearances of the liver tissues constructed under different conditions as follows: non-perfused condition with a normal density of mesenchymal stem cells (MSCs) (**a**), perfused condition with a low density of MSCs (**b**), and perfused condition with the normal density of MSCs (**c**). HUVECs, human umbilical vein endothelial cells. Asterisks indicate the main channels. (**d–f**) HE-stained transverse sections of the liver tissues. (**g–i**) Immunofluorescent images in which cells were stained for nuclei (cyan), CD31 (green), and EpCAM (magenta).

**Figure 4:**
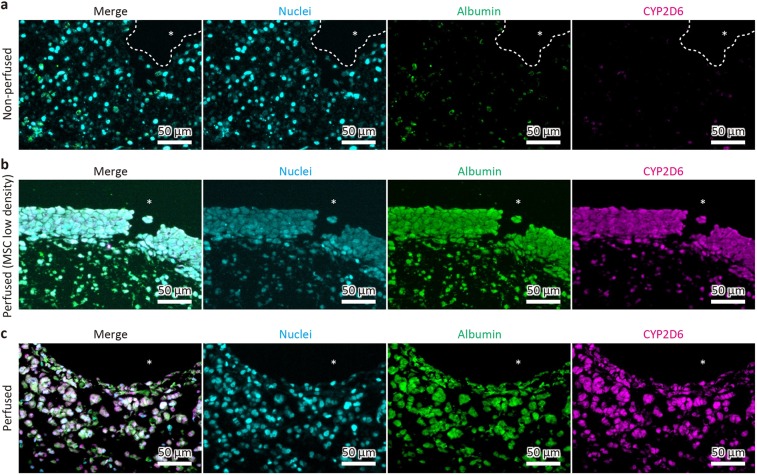
Immunofluorescence of proteins related to liver function in fabricated liver tissues. The non-perfused tissue (**a**), the perfused tissue with a low density of MSCs (**b**), and that with a normal density of MSCs (**c**) were stained for albumin (green) and CYP2D6 (magenta). Asterisks indicate the main channels.

To determine the precise culture conditions for this liver tissue, we prepared tissues using three conditions as follows: a non-perfused condition with a normal density of MSCs (0.5 × 10^7^ cells/mL), a perfused condition with a low density of MSCs (0.05 × 10^7^ cells/mL), and a perfused condition with a normal density of MSCs. As shown in Fig. 3a–c, the shrinkage degree was smallest in the perfused condition with a low density of MSCs, suggesting that MSCs contribute to the shrinkage of tissue composed of cell-populated collagen. Subsequently, haematoxylin and eosin (HE) staining was performed to investigate the tissue structure more precisely (Fig. 3d–f). Of note, the populated cells formed aggregates around the main channel in both perfused conditions but their morphologies appeared different. Compared to that in the perfused conditions, no cell aggregation was observed in the non-perfused condition. These differences were also tested by immunofluorescence for CD31 and EpCAM, which mark HUVECs and HepG2 cells, respectively (Fig. 3g–i, Fig. S1a,b). In the non-perfused condition, no sinusoid-like structures were observed, whereas CD31-positive cells and EpCAM-positive cells were sparsely distributed (Fig. 3g). In the perfused condition with a low density of MSCs, most aggregated cells were stained with EpCAM, whereas CD31-positive cells were scarcely observed (Fig. 3h). In contrast, the CD31-positive area in the perfused condition with a normal density of MSCs was obviously larger than that without perfusion (Fig. 3i, Fig. S1a). The CD31-positive areas formed lumen structures and they appeared to be connected to the main channel as sinusoids. The density of sinusoid-like structures was high around the main channel and gradually decreased with increasing distance from the main channel. Furthermore, the sinusoid-like structures were adjacent to the aggregation of EpCAM-positive HepG2 cells, which was similar to human liver morphology, wherein sinusoids are surrounded by hepatocytes. These results indicate that perfusion is essential for the formation of sinusoid-like structures inside the tissue. Next, we performed immunostaining for albumin and CYP2D6, a major liver protein and typical metabolic enzyme produced in this tissue, respectively (Fig. 4, Fig. S1c,d). Consequently, albumin and CYP2D6 expression was strongly detected in the perfused condition with a normal density of MSCs, compared to that in the non-perfused condition (Fig. 4a,c), indicating that perfusion maintained proper tissue function. Although the expression of albumin and CYP2D6 was also detected in the perfused condition with a low density of MSCs, the expression was intense within approximately 50 μm of the main channel and sparse in the outer region, whereas the expression was maintained in a wider range (100–200 μm) in the perfused condition with a normal density of MSCs (Fig. 4b,c). In addition, it seemed that the rate of decreasing CYP2D6 fluorescence intensity from the main channel was slightly different from that of albumin in the perfused condition with a normal density of MSCs (Fig. S2). This difference was observed within approximately 50 μm of the edge of the channel. According to the results, we compared the non-perfused condition and the perfused condition with normal densities of MSCs in the subsequent experiments.

### Gene expression analysis suggests the upregulation of angiogenesis-related genes after perfusion

**Figure 5:**
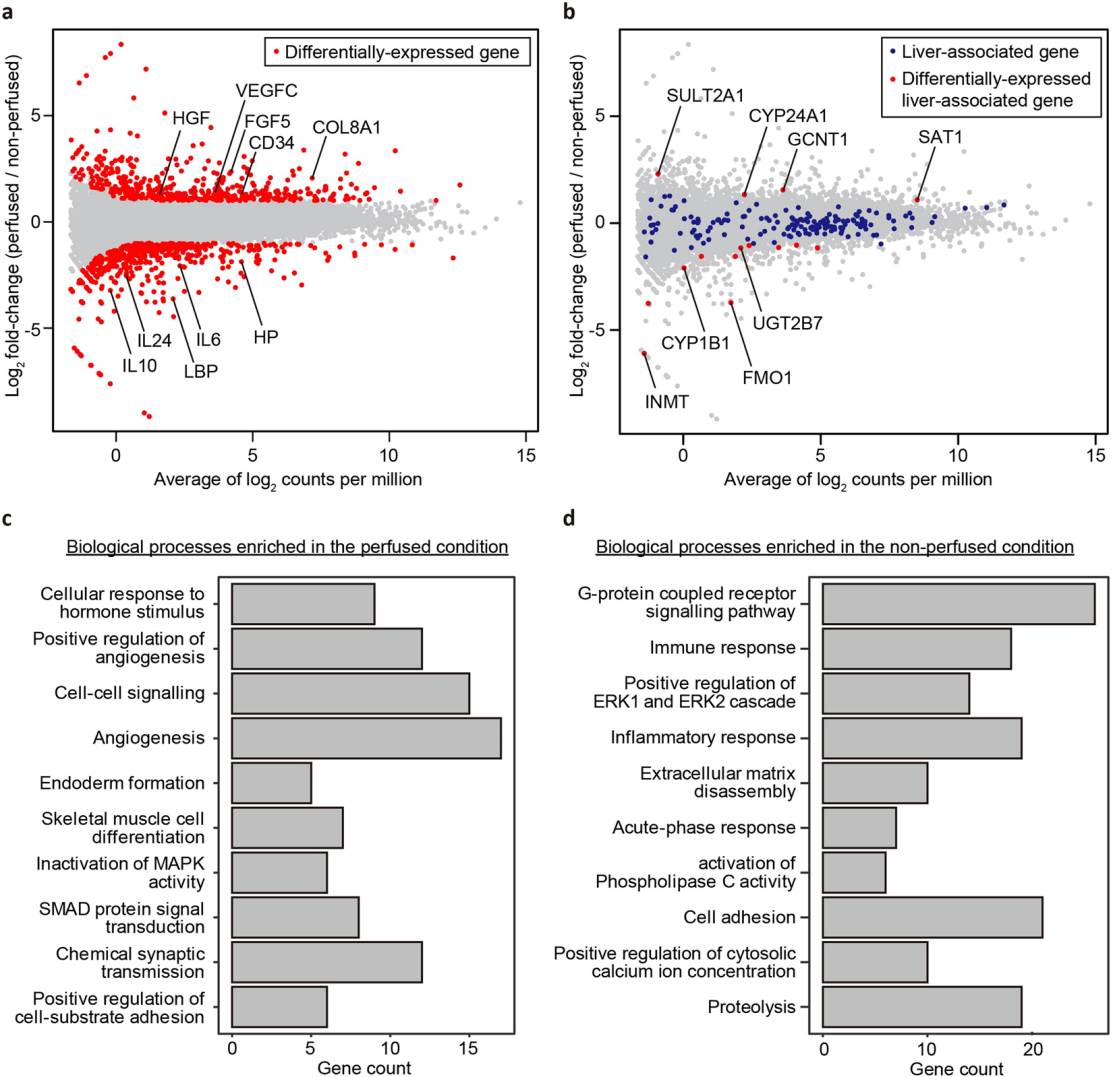
Analysis of global gene expression in the non-perfused and perfused tissues (two independent devices for each condition). (**a**) MA plot, a scatter plot of log_2_ fold-change versus the average of log_2_ counts per million, showing differentially-expressed genes (red points, adjusted *p*-value <0.05 and log_2_ [fold change] ≥1). (**b**) MA plot showing liver-associated genes (blue points) and differentially-expressed genes between tissues (red points). (**c, d**) Top 10 GO terms (biological processes) enriched in the perfused and non-perfused conditions, respectively.

To investigate the influence of perfusion on global gene expression in the liver tissue, we performed RNA-seq at day 7 (Fig. 5). As a result, 954 differentially-expressed genes (adjusted *p*-value <0.05 and log_2_ [fold change] ≥1) were detected (Fig. 5a). Among these, 520 and 434 were enriched in the non-perfused and perfused conditions, respectively. Of note, angiogenesis-associated genes such as *VEGFC*^13^ (an isoform of vascular endothelial growth factor), *HGF*^14^ (hepatocyte growth factor), *FGF5*^15^ (a member of the fibroblast growth factor family), *CD34*^16^ (encoding a transmembrane protein expressed by angiogenic endothelial cells), and *COL8A1*^17^ (encoding type VIII collagen, an ECM protein expressed at angiogenic sites) were upregulated in the perfused condition. These results were consistent with the histological analysis indicating that sinusoid-like structures were observed only in the perfused condition. We also examined the expression of liver-related genes to test the effect of perfusion on liver function. For this, we selected 261 genes including those encoding proteins involved in phase I, II, and III drug metabolism and nuclear receptors that regulate hepatic functions as liver-associated based on previous literature^18–20^. Interestingly, the expression levels of most genes (246 genes) were maintained (Fig. 5b, Fig. S3–S6). In addition, the amount of total RNA extracted from the perfused tissue was 4.6-fold higher than that from non-perfused tissue (Fig. S7). These results indicated that the perfusion maintained not only cell viability but also liver metabolic functions.

To further explore the effect of perfusion, we performed gene ontology (GO) enrichment analysis (Fig. 5c,b, Fig. S8 and S9). As expected, biological processes involved in angiogenesis (GO:0045766, GO:0001525) were enriched in the perfused condition. In contrast, immune or inflammatory responses (GO:0006955, GO:0006954, GO:0006953) were enriched in the non-perfused condition, indicating that the non-perfused condition might result in unhealthy tissue.

### Functional analysis demonstrates substance exchange via the main channel and sinusoid-like structures

**Figure 6:**
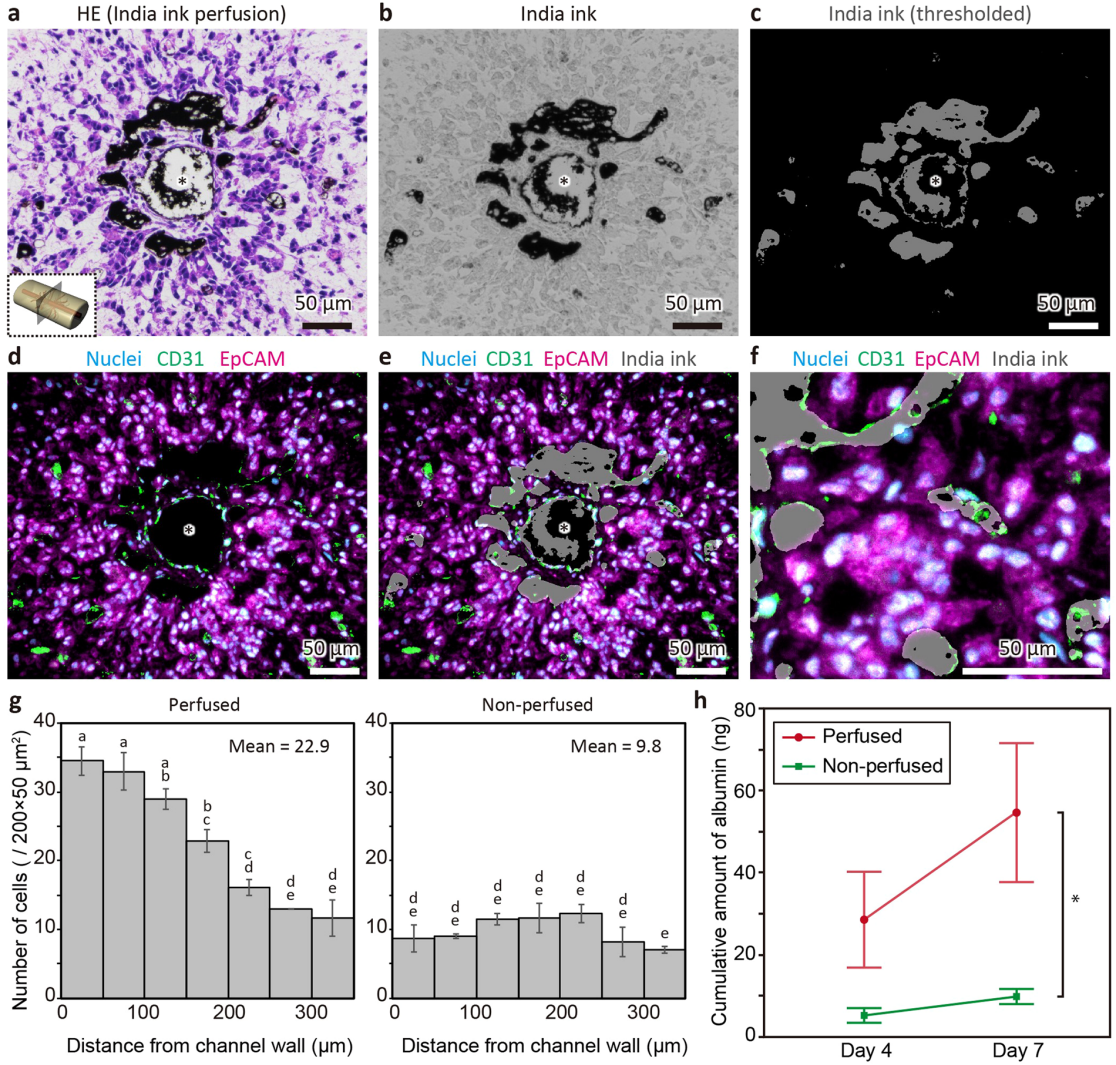
Functional analysis of the main channel and sinusoid-like structures in fabricated liver tissues. (**a**) HE-stained section of the tissue constructed under the perfused condition and subsequently perfused with India ink immediately before fixation. (**b**) Bright field image of the tissue perfused with India ink. (**c**) Image of India ink extracted from the bright field image (**b**) based on the threshold process. (**d**) Immunofluorescent images of (**b**) nuclei (cyan), CD31 (green), and EpCAM (magenta) staining. (**e**) Superimposed image of (**c**) and (**d**). Asterisks indicate the main channels. (**f**) Magnification of (**e**) showing the co-localisation of CD31-positive sinusoid-like structures and India ink. (**g**) Cell distributions around the main channels of the perfused and non-perfused tissues. The data are shown as the mean ± standard error (SE) (n = 3). Statistical analysis was performed by ANOVA, followed by the Tukey-Kramer HSD test. The data were significantly different (*p* < 0.05) if not connected by the same character. (**h**) Cumulative amounts of albumin in the culture medium of the perfused and non-perfused conditions. The data are shown as the mean ± SE (n = 4). Statistical analysis was performed based on an unpaired two-tailed Student’s t-test (*p = 0.039).

To test the function of sinusoid-like structures, we first examined the penetration of India ink from the main channel into the tissue by infusing the ink via the main channel before performing tissue fixation and immunostaining (Fig. 6a–f). As shown in Fig. 6a, the infused ink was observed not only in the main channel but also in the surrounding regions. To analyse the localisation of ink in the tissue more precisely, the ink-positive regions were extracted from the bright field image based on the threshold (Fig. 6b,c) and superimposed with the immunostained image (Fig. 6d–f). Consequently, the superimposed image revealed that the distance of the ink-positive region reached a maximum of 160 μm from the edge of the main channel. Notably, the ink was located in CD31-positive lumen structures (i.e. sinusoid-like structures) but not in CD31-negative and nuclei-negative regions (Fig. S10), indicating that the sinusoid-like structures were connected to the main channel. The connection between the main channel and the sinusoid-like structures was also demonstrated by the immunostaining of serial sections and confocal imaging of the whole tissue using a tissue-clearing technique (Fig. S11 and S12). In both experiments, sinusoid-structures branched from the main channel and forming a network were observed. We also analysed cell distributions around the main channels in the non-perfused and perfused conditions (Fig. 6g). The mean cell number in the region of interest was 22.9 in the perfused condition, which was 2.3-fold higher than that in the non-perfused condition. Notably, the cell density was significantly higher in the region within 200 μm from the main channel compared to that in the non-perfused tissue. Finally, we measured hepatic functions. The levels of albumin secreted into the culture media after 4 and 7 days of culture were quantified by enzyme-linked immunosorbent assay (ELISA) and cumulative amounts of albumin were calculated. As shown in Fig. 6h, the amount of albumin in the perfused condition was 5.5-fold higher than that in the non-perfused condition after 7 days of culture. The activity of cytochrome P450 3 A (CYP3A) was also measured using a luminescence-based assay. As a result, CYP3A activity in the perfused condition was also higher than that in the non-perfused condition (Fig. S13). Taken together, these data suggested that substance exchange, comprising both influx to the tissue and efflux from the tissue, was induced by perfusion via the main channel and sinusoid-like structures.

## Discussion

In this study, we developed a tubular liver tissue containing a perfusable main channel and sinusoid-like structures by combining a perfusion device with a collagen gel populated with different cell lines. We prepared tissues under the non-perfused condition with a normal density of MSCs (0.5 × 10^7^ cells/mL), the perfused condition with a low density of MSCs (0.05 × 10^7^ cells/mL), and the perfused condition with a normal density of MSCs, and found that the shrinkage degree was the lowest in the perfused condition with a low density of MSCs. This result suggested that a normal density of MSCs is preferable because the shrinkage caused by MSCs is thought to be a key factor that induces heterotypic collective behaviours including vascularisation, according to Takebe *et al*.^3,4^. Histological and gene expression analyses revealed that perfusion promotes the vascular formation of sinusoid-like structures and maintains the viability and function of liver tissue. Of note, according to the immunofluorescence analysis, it seemed that the rate of decrease in albumin fluorescence intensity from the main channel was higher than that of CYP2D6, indicating that the zonation of the liver was partially recapitulated due to media supplies; the cellular functions of protein secretion and xenobiotic metabolism in the liver were previously found to be relatively high in the oxygen-rich region and the oxygen-poor region, respectively^21–23^. From the result of gene expression analysis, although most liver-associated genes were not strongly upregulated, the function of the perfused tissue was thought to be enhanced compared to that of the non-perfused tissue when considering whole tissues since the amount of total RNA extracted from the perfused tissue was higher than that obtained without perfusion. Gene expression analysis also revealed that GO terms (biological processes) associated with inflammation were enriched in the non-perfused condition, as compared to expression in the perfused condition. This result indicates the possibility that the live cells in the non-perfused tissue were in an inflammatory state due to stimulation by dead cells^24^ in the deep part of the tissue where oxygen and nutrients were lacking. Substance exchange was also demonstrated by measuring cell density and cumulative amounts of albumin. From the result indicating that cell density was significantly higher around the main channel compared to that in the non-perfused tissue, it was suggested that oxygen and nutrients were supplied by perfusion and also enhanced by the sinusoid-like structures. This speculation was also supported by the result showing that albumin and CYP2D6 expression was maintained in a wider range in the perfused condition with a normal density of MSCs compared to that with a low density of MSCs, which had few sinusoid-like structures. Furthermore, although we used HepG2 cells, HUVECs, and MSCs in this study since they are readily available and suitable for proof-of-concept experiments; our method would be applicable for the general fabrication of 3D tissues augmented with large channels and capillary-like structures, considering that the cells used in this study can be readily changed to other types of parenchymal cells, endothelial cells, and MSCs.

Fabrication methods for large channels in tissue have been proposed; endothelialised channels were previously constructed in heart tissue by dissociating sodium alginate fibres embedded in the tissue^25^, and a tissue populated with dermal fibroblasts containing large channels fabricated by hydrogel 3D printing was also reported^6^. Capillary-like structures have also been fabricated in various ways that harnessed the spontaneous vascularisation of endothelial cells. Specifically, skin tissue prevascularised by adipose-derived cells^26^ and a vascularised liver bud constructed by the self-organisation of iPSC-derived hepatic endoderm cells, MSCs, and HUVECs were reported^11^. Furthermore, combinational approaches have also been reported^27–29^. However, it is still difficult to construct both perfusable large channels and capillary-like structures in tissues populated with a high density of parenchymal cells to our knowledge. A possible reason for this limitation is tissue shrinkage. Further, connections between large channels and the external ports are difficult to maintain when the tissues shrink during cultivation, despite the fact that populating the tissue with MSCs and inducing shrinkage is a promising method to generate dense and vascularised tissue^4^. We overcame this technical difficulty by using a perfusion device with connectors equipped with an anchor and treated with air plasma and fibronectins.

Despite our success, there is still a need to optimise certain culture parameters such as the density and ratio of cells, type and concentration of ECM gel, flow rate of perfusion, and medium composition. In particular, the flow rate might be an important factor since it regulates the rate of substance exchange in the tissue and because the mechanical stress induced by the flow is also involved in growth and maintenance of the liver *in vivo*^30^. In addition, more physiologically-relevant tissues can be obtained using primary cells or iPSC-derived cells instead of cell lines. From this point of view, the liver-like tissue constructed in this study can be considered a hepatic tumour model rather than a normal liver model and thus used for the study of anti-cancer drugs. Furthermore, the tissues can also be enhanced with other non-parenchymal cells such as Kupfer cells and lymphocytes to recreate the immune system and biliary cells for reconstruction of the biliary tree and the transport of bile acid. The source of the endothelial cells might also be important to improve the tissue. By using liver sinusoidal endothelial cells instead of HUVECs, the fenestrated structure of the capillary, the sinusoid-specific morphology, would be formed and contribute to the maintenance of liver function. Moreover, further refinement of the perfusion system is necessary to improve the yield rate since the main channels were occasionally clogged during perfusion, although we performed perfusion for up to 8 days and the duration could be extended to at least 4 weeks like that in other systems^18,31^. Nevertheless, we have focused on demonstrating the feasibility and utility of our method in this study, and further optimisation should be performed with respect to different types of cells. The conditions used in this study could represent a solid foundation for such future investigations.

## Conclusions

We developed a method to fabricate 3D tissues integrated with a perfusable large main channel and capillary-like structures using a perfusion device by constructing a tubular liver tissue consisting of HepG2 cells, HUVECs, and MSCs. This revealed that perfusion via the main channel is essential for the formation of capillary-like structures (i.e. sinusoid-like structures) and the maintenance of cellular functions inside the tissue based on histological analysis and global gene expression analysis. A connection between the main channel and sinusoid-like structures was also shown. Moreover, substance exchange between the tissue and the medium, flowing through the main channel and the sinusoid-like structures, was demonstrated. Therefore, our method can be used to construct 3D tissues enhanced by large perfusable channels and capillary-like structures, which would enable the delivery of test substances into the tissue and the sampling of metabolites from the tissue in basic research, as well as in drug development. It could also contribute to regenerative medicine as large tissues can be maintained by oxygen and nutrient supplies.

## Methods

### Cell culture

HepG2 cells were grown in Dulbecco’s modified Eagle’s medium (DMEM) supplemented with 10% foetal bovine serum (FBS; Thermo Fisher Scientific Inc., Waltham, MA, USA) and 1% penicillin-streptomycin solution (×100; FUJIFILM Wako Pure Chemical Corporation, Osaka, Japan). HUVECs (PromoCell GmbH, Heidelberg, Germany) were cultured in endothelial cell growth medium 2 (EGM-2; PromoCell) supplemented with growth factors included in the medium kit and 1% penicillin-streptomycin solution (×100). Immortalised MSCs (SCRC-4000, ATCC, Manassas, Virginia, USA) were grown in DMEM supplemented with 10% FBS, 1% non-essential amino acids solution (×100) (FUJIFILM Wako Pure Chemical), and 1% penicillin-streptomycin solution (×100). All cells were maintained at 37 °C in a 5% CO_2_ atmosphere.

### Fabrication of the device

The perfusion device was fabricated by a 3D printer (ProJet 3500HD Max, 3D Systems, Inc., Rock Hill, SC, USA) (Fig. S14a). The device was cleaned by an ultrasonic cleaner and sterilised using 70% ethanol. After desiccation in a laminar flow cabinet, the device was treated with air plasma (YHS-R, SAKIGAKE-Semiconductor Co., Ltd., Kyoto, Japan) for 5 minutes and coated with 10 μg/mL bovine fibronectin (Sigma-Aldrich, St. Louis, MO, USA) for 2 hours at 37 °C to increase adhesion of the cells and collagen gel. The device was stored in phosphate buffered saline without Mg^2+^ and Ca^2+^ (PBS) at 4 °C and used for tissue construction within 1 week. Immediately prior to tissue construction, a needle (25 G, Terumo Corporation, Tokyo, Japan) was inserted through the connectors of the device (Fig. S14b-i).

### Tissue construction and culture

HepG2 cells, HUVECs, and MSCs were dissociated from the culture dishes and suspended in 3 mg/mL neutralised type I collagen solution (IAC-50, Koken Co., Tokyo, Japan) at a density of 3 × 10^7^ cells/mL (HepG2), 3 × 10^7^ cells/mL (HUVECs), and 0.5 or 0.05 × 10^7^ cells/mL (MSCs) (Fig. 2a-i, Fig. S14b-ii). After 20 minutes of incubation at 37 °C for gelation, the needle was extracted to form a hollow channel and the device was submerged in medium (Fig. 2a-ii, Fig. S14b-iii). For tissue cultivation, we utilised a 1:1 mixture of DMEM (10% FBS and 1% penicillin-streptomycin) and EGM-2 (growth factors and 1% penicillin-streptomycin) for the non-perfused condition and the perfused condition with a normal density of MSCs (0.5 × 10^7^ cells/mL), and DMEM (10% FBS and 1% penicillin-streptomycin) for the perfused condition with a low density of MSCs (0.05 × 10^7^ cells/mL). The hollow channel was subsequently filled with 100 μL of a HUVEC suspension (4 × 10^6^ cells/mL) using a syringe pump (KD Scientific, Holliston, MA, USA) (Fig. 2a-iii, Fig. S14b-iv). After incubation for 20 minutes, the device was inverted and incubated for another 20 minutes to coat the hollow channel with HUVECs. After 80 minutes, the tissue was perfused with medium at 1 mL/hour using a peristaltic pump (RP-HX01S, Aquatech Co., Ltd., Osaka, Japan) (Fig. 2a-iv,b, Fig. S14b-v). During cultivation, the medium was infused from one of the connectors and drained from the other. The drained medium was mixed with that in the culture dish in which the device was submerged and recirculated by the pump. In the non-perfused condition, the device was only submerged in the medium without circulation by the pump. The flow in the tissue was visualised by infusing India ink (Kuretake Co., Ltd., Nara, Japan) diluted 2:1 in PBS using the syringe pump.

### Histological analysis

The tissues were fixed with 4% paraformaldehyde in phosphate buffer solution (FUJIFILM Wako Pure Chemical) after 4 days of culture and embedded in paraffin wax. The tissues were cut into 5-μm sections and used for HE staining and immunostaining. For HE staining, the sections were dewaxed, rehydrated, and stained with Mayer’s haematoxylin and 0.5% eosin Y ethanol solution (FUJIFILM Wako Pure Chemical). For immunostaining, antigen retrieval was performed by incubating the rehydrated sections in sodium citrate buffer (pH 6.0) for 15 minutes at 121 °C using an autoclave (LSX-300, TOMY SEIKO CO., LTD., Tokyo, Japan). The sections were washed with ultrapure water and blocked with 4% Block Ace (MEGMILK SNOW BRAND Co., Ltd., Tokyo, Japan) in PBS for 20 minutes. Next, the sections were incubated with primary antibodies diluted with 1% Block Ace at 4 °C overnight. The primary antibodies used in this study were as follows: anti-CD31 (1:200, BBA7, R&D Systems, Inc., Minneapolis, MN, USA), anti-EpCAM (1:200, ab71916, Abcam PLC, Cambridge, UK), anti-albumin (1:100, MAB1455, R&D Systems), and anti-CYP2D6 (1:100, AV41675, Sigma-Aldrich). The sections were washed with PBS and incubated with secondary antibodies (Alexa Fluor 488-conjugated anti-mouse or Alexa Fluor 555-conjugated anti-rabbit, Thermo Fisher Scientific) diluted 1:200 in PBS for 1 hour. Subsequently, the sections were counterstained with Hoechst 33342 (Dojindo Molecular Technologies, Kumamoto, Japan).

### Gene expression analysis

Total RNA was isolated from the tissue after 7 days of culture using NucleoSpin RNA (Macherey Nagel GmbH & Co. KG, Duren, Germany). Libraries for RNA-seq were prepared with TruSeq stranded mRNA (Illumina, Inc. San Diego, CA, USA). Sequencing was performed with a NovaSeq 6000 (Illumina). The acquired data from two independent devices for each condition were mapped and quantified using STAR^32^ (2.7.1a) and RSEM^33^ (1.3.1) with hg38 as a reference genome and Ensembl GRCh38 as the gene annotation. Subsequently, differentially-expressed genes were analysed by edgeR^34,35^ (3.24.3) in R^36^ (3.5.1). GO enrichment analysis was performed using the database for annotation, visualisation and integrated discovery (DAVID)^37,38^ via RDAVIDWebService^39^ (1.20.0) and visualised by clusterProfiler^40^ (3.10.1).

### Measurement of cell distribution

To evaluate cell distribution around the main channel, the fixed tissues were embedded in O.C.T. compound (Sakura Finetek Japan Co., Ltd., Tokyo, Japan) and cryosectioned to 7 μm. Images of the sections stained by Hoechst 33342 were analysed with ImageJ software (NIH, Bethesda, MD, USA). The images were converted to binary based on threshold and watershed processes. Then, the number of cells in the upper and lower regions (w × h: 200 × 350 μm) adjacent to the main channel were counted using the analyse particle plugin.

### Quantification of albumin

For albumin quantification in culture supernatant, the culture medium was sampled from the culture dish in which the device was submerged after 4 and 7 days of culture and stored at −80 °C until use. The samples were thawed and assayed with the human albumin ELISA kit (Bethyl Laboratories, Inc., Montgomery, TX, USA).

### Statistical analysis

Cell distribution data were analysed by a one-way analysis of variance (ANOVA) and Tukey-Kramer HSD test using the statistical software JMP 12.2.0 (SAS Institute INC., Cary, NC, USA). Albumin data were analysed by an unpaired Student’s t-test using JMP.

## Supplementary information

Supporting Information.

Movie S1.

## Data Availability

RNA-seq data are available in the DNA Data Bank of Japan (DDBJ) Sequence Read Archive under accession number DRA008972.
