# Supplementary material for: Fabrication of Perfusable Vascular Channels and Capillaries in 3D Liver-like Tissue
Source: Sci Rep. 2020 Apr 14;10:5646. doi: 10.1038/s41598-020-62286-3 (PMC7156376; doi:10.1038/s41598-020-62286-3)
Supplement: Supplementary file 1 — Supporting Information. [file 41598_2020_62286_MOESM1_ESM.pdf]

Supporting Information

Fabrication of Perfusable Vascular Channels and Capillaries in 3D Liver-like Tissue

Nobuhito Mori, Yuka Akagi, Yukiko Imai, Yuzo Takayama and Yasuyuki S. Kida

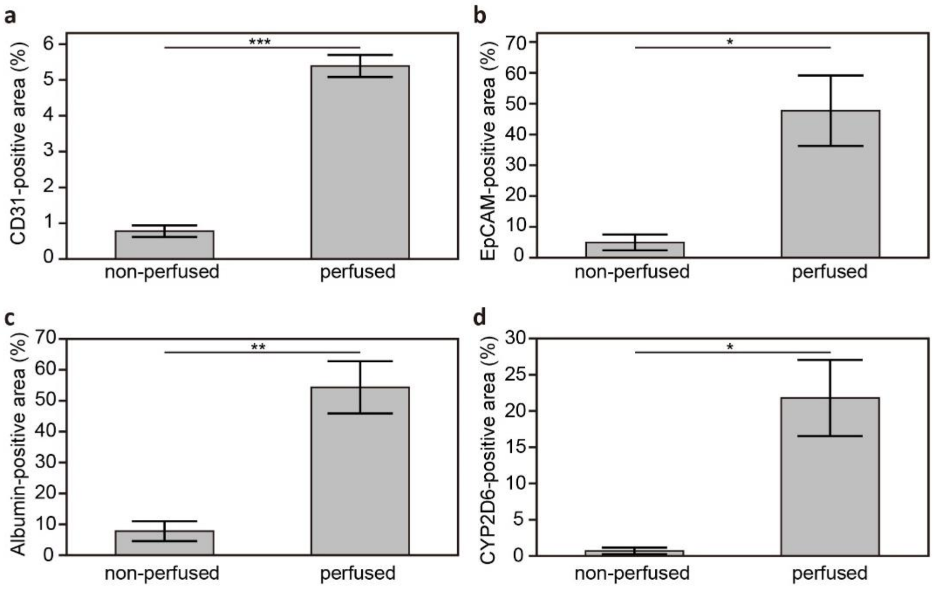

Figure S1. Quantification of immunostained images. The areas of fluorescence corresponding to CD31 (a), EpCAM (b), albumin (c), and CYP2D6 (d) were measured using ImageJ. For each image, the fluorescence-positive pixels were extracted based on a threshold. Subsequently, the fluorescence-positive areas within 200 μm from the main channel and with a width of 100 μm were measured based on four ROIs. The percentage of the fluorescence-positive area in the ROI (200 μm × 100 μm) was calculated. The data are shown as mean ± SE (n = 3). *p < 0.05, **p < 0.01, ***p < 0.001, Student's t-test.

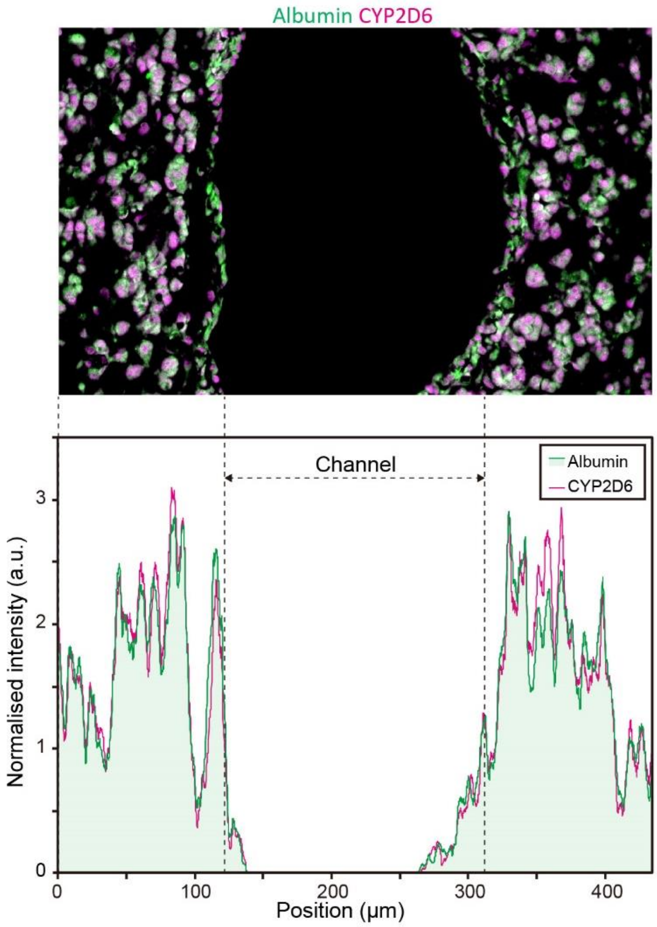

Figure S2. Intensity profile of immunofluorescence for albumin (green line with faint fill) and CYP2D6 (magenta line) in the perfused tissue. The horizontal intensity profile of the upper image was acquired by vertical projection of the intensity using ImageJ. Each intensity value for albumin and CYP2D6 was normalised by each integrated intensity.

EnsemblID	Symbol	logFC	logCPM	FDR
ENSG00000010932	FMO1	-3.72	1.73	3.89e-12
ENSG00000019186	CYP24A1	1.34	2.22	2.50e-05
ENSG00000186204	CYP4F12	-1.56	1.89	4.81e-05
ENSG00000186529	CYP4F3	-1.15	3.47	1.23e-04
ENSG00000106258	CYP3A5	0.88	2.67	6.58e-04
ENSG00000188641	DPYD	0.60	3.74	1.73e-03
ENSG00000138061	CYP1B1	-2.10	0.03	2.62e-03
ENSG00000180432	CYP8B1	-1.05	2.40	4.09e-03
ENSG00000154277	UCHL1	-0.60	3.85	6.81e-03
ENSG00000073756	PTGS2	0.44	4.53	3.03e-02
ENSG00000189221	MAOA	0.32	5.53	3.55e-02
ENSG00000197894	ADH5	-0.28	6.64	5.11e-02
ENSG00000112294	ALDH5A1	-0.31	4.71	7.05e-02
ENSG00000131781	FMO5	1.05	0.03	8.02e-02
ENSG00000095596	CYP26A1	1.26	-0.49	1.06e-01
ENSG00000072506	HSD17B10	-0.26	4.73	1.20e-01
ENSG00000072210	ALDH3A2	-0.24	5.17	1.43e-01
ENSG00000140465	CYP1A1	-0.94	0.74	1.63e-01
ENSG00000140459	CYP11A1	-0.54	1.50	2.03e-01
ENSG00000167600	CYP2S1	0.31	4.60	2.20e-01
ENSG00000135929	CYP27A1	-0.22	5.17	2.34e-01
ENSG00000118514	ALDH8A1	-1.58	-1.37	2.44e-01
ENSG00000139684	ESD	0.18	6.50	2.69e-01
ENSG00000137124	ALDH1B1	-0.20	4.95	2.78e-01
ENSG00000143149	ALDH9A1	0.20	4.49	3.11e-01
ENSG00000186104	CYP2R1	-0.60	0.47	3.24e-01
ENSG00000118939	UCHL3	0.18	4.29	3.86e-01
ENSG00000164904	ALDH7A1	0.16	5.65	3.86e-01
ENSG00000165092	ALDH1A1	0.24	3.04	4.17e-01
ENSG00000198099	ADH4	0.62	-0.41	4.91e-01
ENSG00000073067	CYP2W1	-0.13	5.26	4.92e-01
ENSG00000231852	CYP21A2	-0.37	0.90	5.26e-01
ENSG00000184254	ALDH1A3	0.22	2.85	5.56e-01
ENSG00000160870	CYP3A7	-0.48	-0.11	5.56e-01
ENSG00000159423	ALDH4A1	0.11	5.98	5.64e-01
ENSG00000111012	CYP27B1	-0.43	0.33	6.23e-01
ENSG00000137869	CYP19A1	-0.18	2.15	6.77e-01
ENSG00000069535	MAOB	0.10	4.10	6.99e-01
ENSG00000100197	CYP2D6	0.29	0.29	7.20e-01
ENSG00000111275	ALDH2	-0.07	5.89	7.21e-01
ENSG00000006534	ALDH3B1	-0.09	4.88	7.29e-01
ENSG00000076258	FMO4	-0.44	-1.13	7.69e-01
ENSG00000003137	CYP26B1	-0.20	0.98	7.89e-01
ENSG00000119711	ALDH6A1	-0.07	3.96	8.14e-01
ENSG00000128918	ALDH1A2	0.17	1.29	8.22e-01
ENSG00000172955	ADH6	0.14	1.25	8.70e-01
ENSG00000095303	PTGS1	0.03	5.03	9.17e-01
ENSG00000158125	XDH	-0.11	-1.17	1.00e+00
ENSG00000130649	CYP2E1	0.02	0.53	1.00e+00
ENSG00000197408	CYP2B6	-0.07	-1.08	1.00e+00
ENSG00000148795	CYP17A1	-0.02	-0.92	1.00e+00
ENSG00000114771	AADAC			
ENSG00000187758	ADH1A			
ENSG00000196616	ADH1B			
ENSG00000248144	ADH1C			
ENSG00000196344	ADH7			
ENSG00000108602	ALDH3A1			
ENSG00000132746	ALDH3B2			
ENSG00000170835	CEL			
ENSG00000160882	CYP11B1			
ENSG00000179142	CYP11B2			
ENSG00000140505	CYP1A2			
ENSG00000187553	CYP26C1			
ENSG00000197838	CYP2A13			
ENSG00000108242	CYP2C18			
ENSG00000165841	CYP2C19			
ENSG00000138115	CYP2C8			
ENSG00000138109	CYP2C9			
ENSG00000197446	CYP2F1			
ENSG00000160868	CYP3A4			
ENSG00000021461	CYP3A43			
ENSG00000187048	CYP4A11			
ENSG00000162365	CYP4A22			
ENSG00000142973	CYP4B1			
ENSG00000171903	CYP4F11			
ENSG00000186115	CYP4F2			
ENSG00000186526	CYP4F8			
ENSG00000167910	CYP7A1			
ENSG00000172817	CYP7B1			
ENSG00000100867	DHRS2			
ENSG00000094963	FMO2			
ENSG00000007933	FMO3			
ENSG00000145649	GZMA			
ENSG00000100453	GZMB			

Figure S3. Fold-changes and expression levels of genes related to phase I drug metabolisation. Undetected genes are shown without data. FC: fold-change, CPM: counts per million, FDR: false discovery rate.

EnsemblID	Symbol	logFC	logCPM	FDR
ENSG00000166741	NNMT	-0.98	7.19	4.15e-16
ENSG00000187210	GCNT1	1.55	3.62	1.76e-12
ENSG00000130066	SAT1	1.09	8.51	3.18e-11
ENSG00000213366	GSTM2	-1.03	4.12	2.95e-10
ENSG00000148154	UGCG	0.76	5.64	1.67e-09
ENSG00000008394	MGST1	-0.63	6.00	1.77e-07
ENSG00000257594	GALNT4	-1.16	4.88	1.90e-07
ENSG00000123983	ACSL3	0.54	6.40	8.97e-06
ENSG00000068366	ACSL4	0.48	7.03	8.54e-05
ENSG00000151726	ACSL1	-0.52	6.36	8.79e-05
ENSG00000171234	UGT2B7	-1.16	2.10	1.15e-04
ENSG00000241644	INMT	-6.10	-1.42	1.13e-03
ENSG00000181019	NQO1	-0.56	7.03	1.30e-03
ENSG00000134201	GSTM5	-3.75	-1.27	2.28e-03
ENSG00000005187	ACSM3	-0.64	3.91	3.25e-03
ENSG00000143819	EPHX1	-0.47	4.86	4.15e-03
ENSG00000124588	NQO2	-0.47	4.46	4.30e-03
ENSG00000085998	POMGNT1	-0.37	5.77	5.38e-03
ENSG00000170899	GSTA4	-0.47	4.17	1.52e-02
ENSG00000198203	SULT1C2	-0.75	2.34	1.52e-02
ENSG00000131446	MGAT1	0.36	7.48	1.87e-02
ENSG00000136881	BAAT	0.41	5.32	1.94e-02
ENSG00000105398	SULT2A1	2.30	-0.92	2.03e-02
ENSG00000214435	AS3MT	-0.51	3.66	3.65e-02
ENSG00000172831	CES2	-0.36	4.85	4.22e-02
ENSG00000143198	MGST3	-0.29	5.51	4.32e-02
ENSG00000148344	PTGES	-0.36	5.39	5.38e-02
ENSG00000171097	KYAT1	-0.32	4.02	1.03e-01
ENSG00000197448	GSTK1	-0.21	5.73	1.60e-01
ENSG00000197165	SULT1A2	-0.53	2.81	1.67e-01
ENSG00000171428	NAT1	-0.77	0.93	2.36e-01
ENSG00000196502	SULT1A1	-0.23	5.81	2.77e-01
ENSG00000172828	CES3	0.26	3.96	2.79e-01
ENSG00000128311	TST	-0.20	4.74	2.94e-01
ENSG00000173597	SULT1B1	0.77	-0.28	3.03e-01
ENSG00000172482	AGXT	0.35	3.35	3.13e-01
ENSG00000130005	GAMT	-0.18	4.86	3.31e-01
ENSG00000141429	GALNT1	0.15	6.18	3.55e-01

EnsemblID	Symbol	logFC	logCPM	FDR
ENSG00000168282	MGAT2	0.18	4.00	4.09e-01
ENSG00000148834	GSTO1	-0.13	6.56	4.22e-01
ENSG00000120915	EPHX2	-0.24	3.30	4.51e-01
ENSG00000196433	ASMT	0.79	-0.81	4.77e-01
ENSG00000150540	HNMT	-0.16	5.39	4.82e-01
ENSG00000168765	GSTM4	-0.19	3.84	4.85e-01
ENSG00000084207	GSTP1	0.10	5.82	5.61e-01
ENSG00000137364	TPMT	-0.13	4.07	6.21e-01
ENSG00000198075	SULT1C4	0.12	3.13	7.52e-01
ENSG00000173418	NAA20	-0.06	5.14	7.88e-01
ENSG00000085871	MGST2	0.06	5.25	8.06e-01
ENSG00000088002	SULT2B1	0.21	0.04	8.46e-01
ENSG00000134202	GSTM3	-0.04	5.22	8.53e-01
ENSG00000066813	ACSM2B	-0.30	-0.99	8.72e-01
ENSG00000244038	DDOST	0.03	9.05	8.89e-01
ENSG00000093010	COMT	0.02	6.50	9.33e-01
ENSG00000166743	ACSM1	0.24	-1.22	9.41e-01
ENSG00000141744	PNMT	0.10	0.54	9.53e-01
ENSG00000065621	GSTO2	-0.02	4.14	9.71e-01
ENSG00000124713	GNMT	0.04	0.38	1.00e+00
ENSG00000135220	UGT2A3	0.03	1.09	1.00e+00
ENSG00000147119	CHST7	0.01	1.39	1.00e+00
ENSG00000129673	AANAT			
ENSG00000198848	CES1			
ENSG00000159398	CES5A			
ENSG00000149124	GLYAT			
ENSG00000243955	GSTA1			
ENSG00000174156	GSTA3			
ENSG00000182793	GSTA5			
ENSG00000277656	GSTT1			
ENSG00000156006	NAT2			
ENSG00000196228	SULT1C3			
ENSG00000109193	SULT1E1			
ENSG00000130540	SULT4A1			
ENSG00000138068	SULT6B1			
ENSG00000241635	UGT1A1			
ENSG00000244474	UGT1A4			
ENSG00000241119	UGT1A9			
ENSG00000173610	UGT2A1			
ENSG00000109181	UGT2B10			
ENSG00000197888	UGT2B17			
ENSG00000135226	UGT2B28			
ENSG00000156096	UGT2B4			
ENSG00000145626	UGT3A1			
ENSG00000174607	UGT8			

Figure S4. Fold-changes and expression levels of genes related to phase II drug metabolisation. Undetected genes are shown without data. FC: fold-change, CPM: counts per million, FDR: false discovery rate.

EnsemblID	Symbol	logFC	logCPM	FDR
ENSG00000117394	SLC2A1	0.85	11.66	2.87e-09
ENSG00000059804	SLC2A3	0.73	11.04	1.47e-08
ENSG00000017483	SLC38A5	-0.57	6.92	5.22e-06
ENSG00000134294	SLC38A2	0.69	10.25	8.30e-06
ENSG00000168003	SLC3A2	0.59	6.76	1.83e-05
ENSG00000119688	ABCD4	-0.51	5.15	2.23e-04
ENSG00000155465	SLC7A7	-0.48	5.74	5.40e-04
ENSG00000165637	VDAC2	0.47	8.24	1.10e-03
ENSG00000174640	SLCO2A1	0.95	1.93	2.03e-03
ENSG00000185883	ATP6V0C	0.40	6.28	3.97e-03
ENSG00000165269	AQP7	-1.56	0.66	3.99e-03
ENSG00000123191	ATP7B	-0.52	4.58	4.32e-03
ENSG00000156222	SLC28A1	-0.96	2.56	4.44e-03
ENSG00000168394	TAP1	-0.53	4.22	6.39e-03
ENSG00000103257	SLC7A5	0.34	8.09	1.31e-02
ENSG00000108846	ABCC3	0.34	6.33	2.29e-02
ENSG00000141526	SLC16A3	0.30	9.15	2.49e-02
ENSG00000103222	ABCC1	0.25	7.48	7.58e-02
ENSG00000088386	SLC15A1	-1.27	-0.32	7.71e-02
ENSG00000213585	VDAC1	0.24	8.33	8.18e-02
ENSG00000013364	MVP	-0.26	7.67	9.78e-02
ENSG00000154258	ABCA9	-1.15	0.30	1.03e-01
ENSG00000146477	SLC22A3	-0.38	2.97	1.38e-01
ENSG00000179869	ABCA13	1.25	-0.67	1.53e-01
ENSG00000101986	ABCD1	0.22	5.21	1.56e-01
ENSG00000173638	SLC19A1	-0.22	4.64	2.31e-01
ENSG00000136868	SLC31A1	0.21	5.57	2.38e-01
ENSG00000023839	ABCC2	-0.21	8.31	2.43e-01
ENSG00000143921	ABCG8	-0.70	0.52	2.57e-01
ENSG00000085563	ABCB1	0.43	1.59	3.00e-01
ENSG00000114770	ABCC5	-0.18	5.49	3.43e-01
ENSG00000204267	TAP2	-0.26	3.80	3.49e-01
ENSG00000151012	SLC7A11	-0.31	4.43	3.51e-01
ENSG00000137491	SLCO2B1	-0.18	4.68	3.67e-01
ENSG00000092068	SLC7A8	0.31	2.18	3.94e-01
ENSG00000021488	SLC7A9	1.09	-1.17	4.03e-01
ENSG00000147100	SLC16A2	0.31	2.24	4.32e-01
ENSG00000175003	SLC22A1	-0.88	-1.17	4.72e-01

EnsemblID	Symbol	logFC	logCPM	FDR
ENSG00000154265	ABCA5	-0.15	5.27	4.77e-01
ENSG00000163406	SLC15A2	0.61	-0.36	4.81e-01
ENSG00000112759	SLC29A1	0.11	6.20	5.21e-01
ENSG00000137204	SLC22A7	-0.88	-1.17	5.37e-01
ENSG00000155380	SLC16A1	-0.10	6.55	5.90e-01
ENSG00000165240	ATP7A	0.13	4.37	6.46e-01
ENSG00000117528	ABCD3	0.09	5.54	6.47e-01
ENSG00000103064	SLC7A6	0.08	6.61	6.56e-01
ENSG00000124574	ABCC10	0.09	5.07	6.58e-01
ENSG00000107331	ABCA2	-0.08	6.82	6.62e-01
ENSG00000125257	ABCC4	-0.09	4.85	6.69e-01
ENSG00000115657	ABCB6	-0.07	6.42	7.02e-01
ENSG00000176463	SLCO3A1	-0.33	0.39	7.07e-01
ENSG00000240583	AQP1	0.16	4.26	7.12e-01
ENSG00000101187	SLCO4A1	0.10	7.65	7.12e-01
ENSG00000004864	SLC25A13	0.07	4.76	7.57e-01
ENSG00000121270	ABCC11	0.15	1.50	8.00e-01
ENSG00000165029	ABCA1	-0.07	6.61	8.11e-01
ENSG00000174669	SLC29A2	0.19	0.53	8.14e-01
ENSG00000117479	SLC19A2	0.06	4.53	8.35e-01
ENSG00000084453	SLCO1A2	-0.42	-1.42	8.63e-01
ENSG00000198691	ABCA4	0.13	1.62	8.72e-01
ENSG00000204574	ABCF1	-0.03	6.33	8.89e-01
ENSG00000167972	ABCA3	0.03	4.99	9.29e-01
ENSG00000163581	SLC2A2			
ENSG00000138079	SLC3A1			
ENSG00000100170	SLC5A1			
ENSG00000005471	ABCB4			
ENSG00000125255	SLC10A2			
ENSG00000100652	SLC10A1			
ENSG00000112499	SLC22A2			
ENSG00000073734	ABCB11			
ENSG00000137860	SLC28A2			
ENSG00000149452	SLC22A8			
ENSG00000197901	SLC22A6			
ENSG00000118777	ABCG2			
ENSG00000134538	SLCO1B1			
ENSG00000100191	SLC5A4			
ENSG00000111700	SLCO1B3			
ENSG00000103569	AQP9			
ENSG00000197506	SLC28A3			
ENSG00000135917	SLC19A3			
ENSG00000140798	ABCC12			
ENSG00000149742	SLC22A9			
ENSG00000144452	ABCA12			
ENSG00000004846	ABCB5			

Figure S5. Fold-changes and expression levels of genes related to phase III drug metabolisation. Undetected genes are shown without data. FC: fold-change, CPM: counts per million, FDR: false discovery rate.

EnsemblID	Symbol	logFC	logCPM	FDR
ENSG00000131910	NR0B2	0.73	4.19	3.20e-05
ENSG00000111424	VDR	-0.91	3.31	9.59e-04
ENSG00000163631	ALB	0.59	2.40	7.45e-02
ENSG00000101076	HNF4A	-0.17	6.45	2.93e-01
ENSG00000012504	NR1H4	-0.58	0.61	4.27e-01
ENSG00000106546	AHR	-0.16	4.29	4.56e-01
ENSG00000131408	NR1H2	0.13	5.10	4.73e-01
ENSG00000025434	NR1H3	0.15	4.48	4.74e-01
ENSG00000144852	NR1I2	0.05	3.43	9.05e-01
ENSG00000143257	CAR			

Figure S6. Fold-changes and expression levels of albumin gene and nuclear receptor genes related to liver function. Undetected genes are shown without data. FC: fold-change, CPM: counts per million, FDR: false discovery rate.

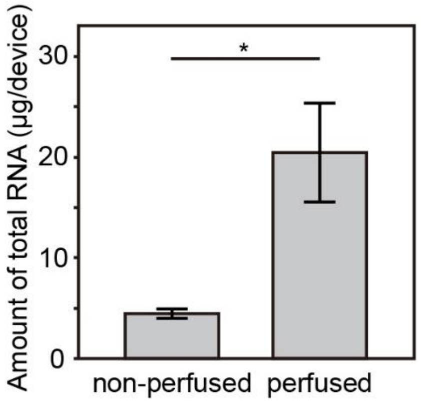

Figure S7. Amount of total RNA. The data are shown as mean ± SE (n = 4 for non-perfused, and n = 3 for perfused). *$p < 0.05$, Student's t-test.

GO ID	Term	p.adjust	Genes
GO:0032870	cellular response to hormone stimulus	8.88E-03	SIK1, JUN, SLIT2, FOS, ROBO2, SIK1B, OXTR, DUSP1, ASNS
GO:0045766	positive regulation of angiogenesis	1.87E-02	SERPINE1, TERT, NODAL, VEGFC, PRKD1, CCBE1, DDAH1, FLT1, CD34, CXCL8, ADM, HGF
GO:0007267	cell-cell signalling	2.46E-02	ADRA1B, IHH, IL11, SSTR1, ADRA2C, NTF3, GJA3, ZYX, CTGF, CYR61, FGF5, GDF15, BARX1, ADM, BDNF
GO:0001525	angiogenesis	4.39E-02	NRXN3, SERPINE1, ANXA2, NDNF, PRKD1, VEGFC, KLF5, S1PR1, TNFRSF12A, JUN, SAT1, CTGF, COL8A1, ITGA5, LEP, FLT1, CXCL8
GO:0001706	endoderm formation	4.44E-02	DUSP5, NOG, DUSP4, DUSP2, DUSP1
GO:0035914	skeletal muscle cell differentiation	1.05E-01	KLF5, ANKRD1, ATF3, HIVEP3, FOS, ASB2, EGR1
GO:0000188	inactivation of MAPK activity	1.20E-01	DUSP10, DUSP5, DUSP8, DUSP4, DUSP2, DUSP1
GO:0060395	SMAD protein signal transduction	1.24E-01	ATOH8, JUN, NODAL, BTBD11, FOS, AFP, GDF15, SMAD9
GO:0007268	chemical synaptic transmission	1.45E-01	CHRNA4, PLP1, GPR176, CACNB4, KCNN1, GRIA1, CACNA1G, KCNQ5, GABBR2, GPR1, SSTR1, OPRD1
GO:0010811	positive regulation of cell-substrate adhesion	1.92E-01	NDNF, COL8A1, CCDC80, ITGA5, CYR61, ABI3BP

Figure S8. Top 10 biological processes enriched in the perfused condition.

GO ID	Term	p.adjust	Genes
GO:0007186	G-protein coupled receptor signalling pathway	1.50E-07	AKR1C3, ADGRV1, ACKR4, GPR119, PTGIR, NMUR1, GPR68, PTGER1, ADGRF4, AKR1C2, ADGRF2, CCR10, CCR7, CX3CL1, RGS16, EDNRA, SFRP2, MRGPRF, C5, CCL14, CCL5, RGS6, ADGRD1, ADGRE2, ADCYAP1R1, GPR153
GO:0006955	immune response	9.55E-04	CD36, OAS2, SPN, ACKR4, IL10, AIRE, TNFSF12-TNFSF13, CCR10, CCR7, CX3CL1, IL24, IFI6, IL1R1, CCL14, C1R, CCL5, IL6, IL1RL1
GO:0070374	positive regulation of ERK1 and ERK2 cascade	1.26E-03	CD36, S100A7, HTR2A, FGA, C5AR2, FGB, FGG, CCR7, CX3CL1, CCL14, EPO, CCL5, GPNMB, IL6
GO:0006954	inflammatory response	1.78E-03	AL049839.2, BMPR1B, C5AR2, PTGIR, PTGER1, GPR68, IL10, CCR7, IL24, CDO1, C5, SCG2, CCL14, CCL5, SPP1, IL6, TNFAIP6, ADGRE2, S100A9
GO:0022617	extracellular matrix disassembly	2.05E-03	MMP20, MMP2, MMP9, MMP13, MMP1, A2M, SPP1, ADAMTS5, CTSK, DCN
GO:0006953	acute-phase response	2.74E-03	AL049839.2, HP, ITIH4, LBP, EPO, TFR2, IL6
GO:0007202	activation of phospholipase C activity	4.88E-03	EDNRA, HTR2A, NMUR1, ARHGAP6, PLCB2, ADCYAP1R1
GO:0007155	cell adhesion	5.20E-03	CD36, AC068234.1, SELPLG, COMP, CYP1B1, THBS4, CX3CL1, CDH15, DPT, SELL, SPON2, LAMA4, SPP1, ITGB3, MFAP4, GPNMB, HABP2, CD24, ADAM12, SEMA5A, TNFAIP6, ADGRE2
GO:0007204	positive regulation of cytosolic calcium ion concentration	6.68E-03	CD36, EDNRA, C5AR2, PTGIR, GIPR, PTGER1, C1QTNF1, CD24, CCR10, CCR7
GO:0006508	proteolysis	1.36E-02	MMP13, MMP1, PCSK1, ADAMTS14, ADAMTS5, CTSK, C1S, ADAMTS13, MMP2, MMP20, MMP9, PAPPA, CPZ, ADAM33, C1R, NAALADL1, HABP2, ADAM12, CTSF

Figure S9. Top 10 biological processes enriched in the non-perfused condition.

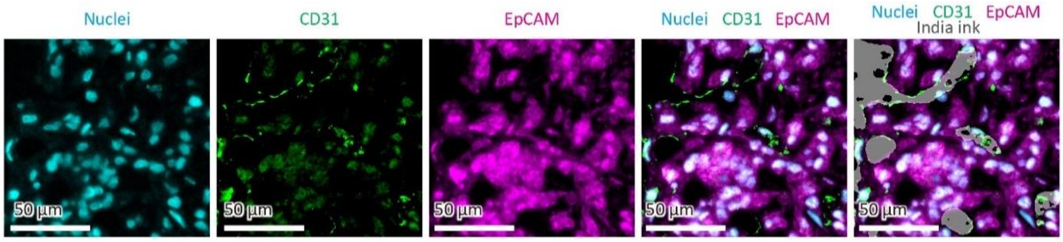

Figure S10. Immunostaining of the tissue infused with India ink. The experiment was performed as follows. After cultivation in the perfused condition, the tissue was infused with India ink via the main channel immediately before fixation. Subsequently, the tissue was embedded in paraffin, sectioned, and immunostained with anti-CD31 and anti-EpCAM antibodies. India ink extracted from the bright field image based on a threshold was superimposed with the fluorescent image.

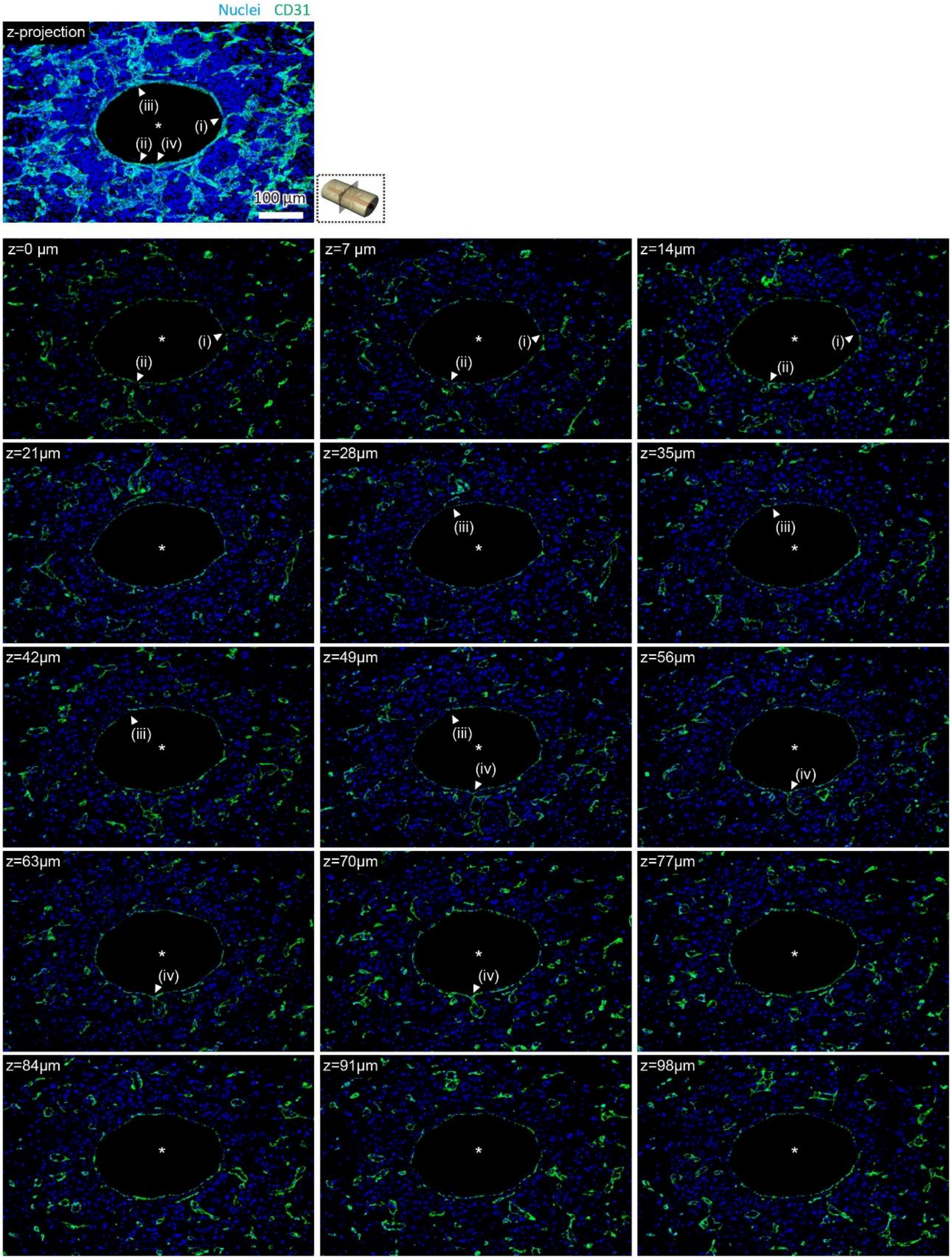

Figure S11. Immunostaining of serial sections. The main channel and branches of the sinusoid-like structures from the main channel are indicated by an asterisk and arrowheads, respectively. The experiment was performed as follows. Serial sections of

the tissue cultured in the perfused condition were made at a thickness of 7 μm. The sections were stained using an anti-CD31 antibody and ImmPRESS Reagent and ImmPACT DAB (Vector Laboratories, Inc., Burlingame, CA, USA) according to the manufacturer's instructions, and counterstained with haematoxylin. The images of the immunostained sections were processed with ImageJ. First, the images were aligned using the StackReg plug-in. Subsequently, the colours of nuclei and CD31 were extracted by colour deconvolution and converted to pseudo colours. Although EGM was used as a culture medium for this experiment, it would not significantly affect the results.

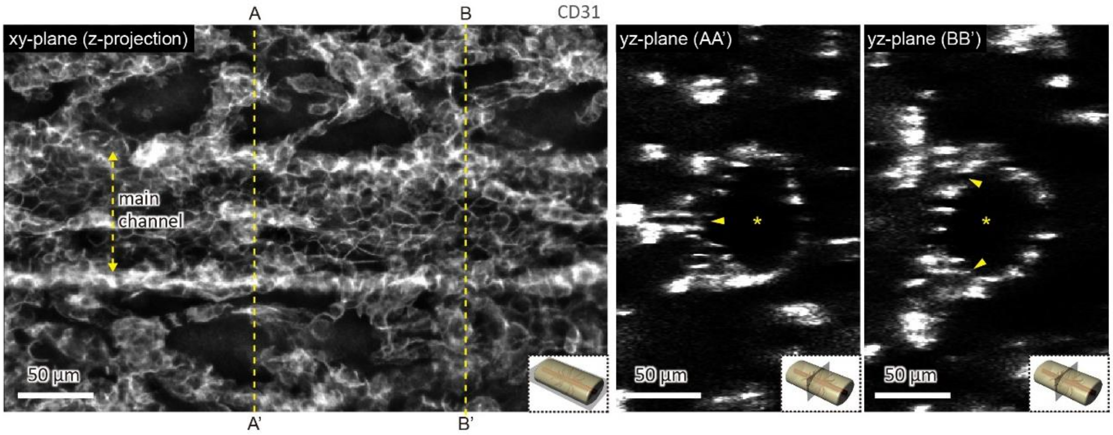

Figure S12. Three-dimensional confocal image of the whole tissue. The main channel is indicated by a dotted line with arrow heads in the xy-plane and asterisks in the yz-planes. The branches of sinusoid-like structures from the main channel are indicated by arrowheads in the yz-planes. The experiment was performed as follows. The tissue cultured in the perfused condition was fixed, immunostained for CD31, and cleared with CUBIC (Tokyo Chemical Industry Co., Ltd., Tokyo, Japan) according to the manufacturer's instruction since the tissue was too thick to observe. The tissue was observed with a confocal microscope. The acquired image was deconvolved with the Richardson-Lucy algorithm (20 iterations) using the DeconvolutionLab plug-in of ImageJ to improve the contrast and resolution. Although EGM was used as a culture medium and normal MSCs were used instead of immortalised MSCs for this experiment, this would not significantly affect the results.

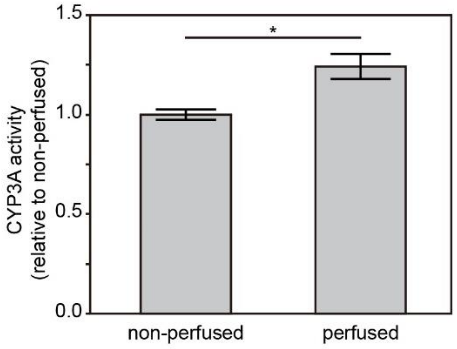

Figure S13. Measurement of CYP3A activity in tissues cultured in non-perfused and perfused conditions. CYP3A activity was measured based on the P450-Glo Assay (Promega, Madison, WI, USA) according to the manufacturer's instruction. Briefly, the culture medium was changed to that containing luciferin-PFBE at day 2. Subsequently, the tissue was perfused with medium in the perfused condition, whereas the tissue was only submerged in the medium in the non-perfused condition. The medium in the culture dish was sampled at day 7 and mixed with luciferin detection reagent. The luminescence intensity, which represents CYP3A activity, was measured with a luminometer. The data are shown as the mean ± SE (n = 3). *$p < 0.05$, Student's t-test.

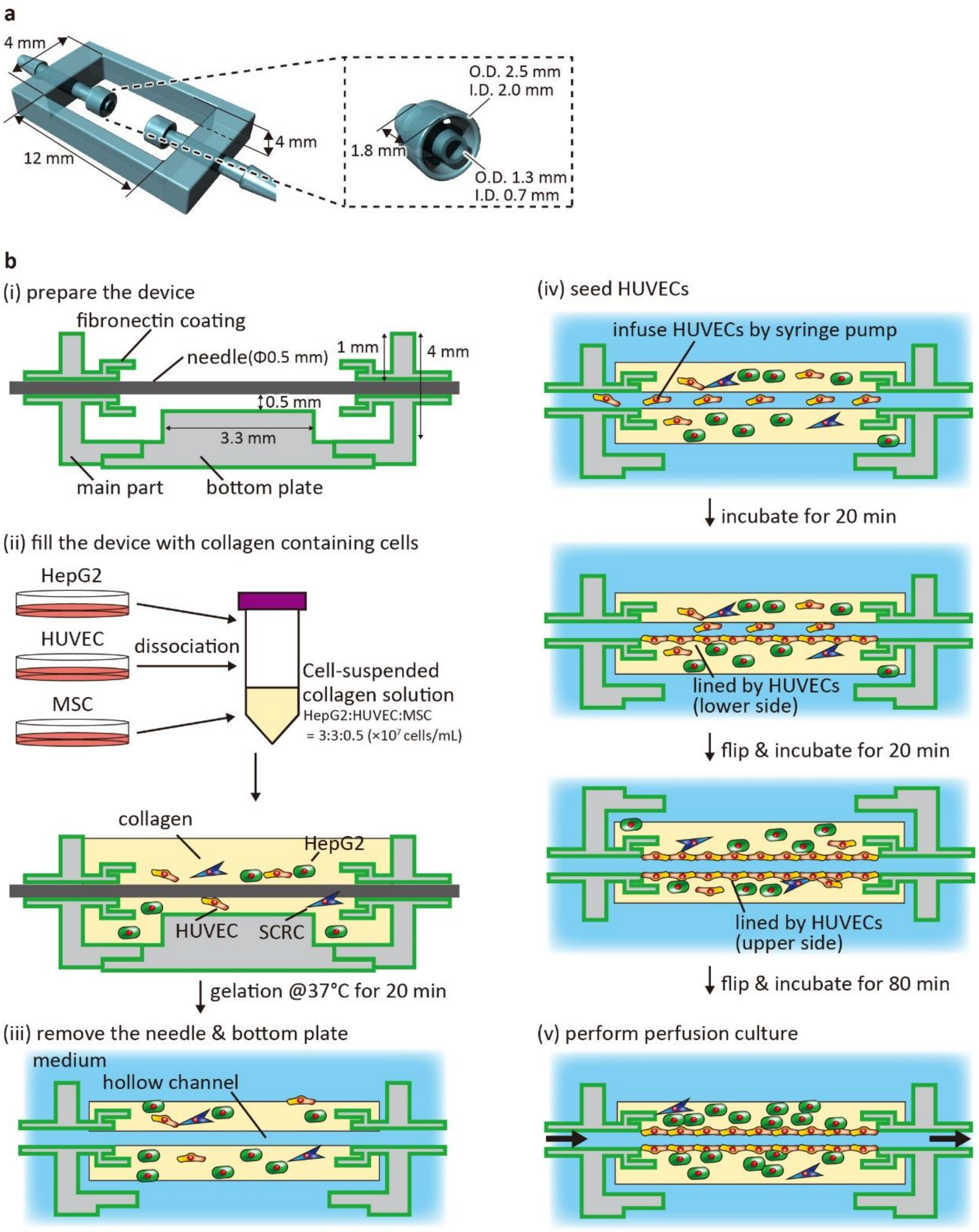

Figure S14. Details of tissue construction. (a) Dimensions of the device. (b) Step-by-step procedure: (i) The device was treated with air plasma and coated with fibronectin. The bottom plate was set for sealing. A needle (25 G) was inserted through the connectors. (ii) Cells were dissociated from the culture dishes and suspended in neutralised type I

collagen solution. The cell-suspended collagen solution was poured into the device and incubated for 20 minutes at 37 °C for gelation. (iii) The needle was removed to make a hollow channel. The bottom plate was also removed to expose the tissue to the medium. The device was submerged in the medium. (iv) The suspension of HUVECs was infused into the hollow channel using a syringe pump. The device was incubated in a CO_2 incubator for 20 minutes to cover the lower side of the channel with HUVECs. The device was inverted again and incubated for another 80 minutes for the further adhesion of HUVECs. (v) Perfusion is started using a peristaltic pump.
